# Assessing current and future areas of ecological suitability for *Lutzomyia shannoni* in North America

**DOI:** 10.1186/s13071-025-06781-4

**Published:** 2025-04-25

**Authors:** Sydney DeWinter, Grace K. Nichol, Christopher Fernandez-Prada, Amy L. Greer, J. Scott Weese, Katie M. Clow

**Affiliations:** 1 Department of Population Medicine, Ontario Veterinary College, University of Guelph, Guelph, ON Canada; 2 Department of Pathology and Microbiology, Faculty of Veterinary Medicine, University of Montreal, Saint-Hyacinthe, QC Canada; 3 Department of Biology, Trent University, Peterborough, ON Canada; 4 Department of Pathobiology, Ontario Veterinary College, University of Guelph, Guelph, ON Canada

**Keywords:** *Lutzomyia shannoni*, *Leishmania* spp., Vesicular stomatitis virus, Climate change, Range expansion, Ecological suitability, Ecological niche modeling

## Abstract

**Background:**

In the Americas, sand flies of the *Lutzomyia* genus are the vectors of pathogens of human and animal health significance. *Lutzomyia shannoni* is suspected to transmit vesicular stomatitis virus, along with *Leishmania mexicana* and *Leishmania infantum* (causative agents of leishmaniases). Despite the suspected vector potential of *Lu. shannoni*, significant knowledge gaps remain, including how ongoing climate changes could facilitate their range expansion. The objectives of this study were to predict the current and future ecological suitability of regions across North America for *Lu. shannoni* and to identify variables driving ecological suitability.

**Methods:**

Occurrence records were obtained from the Global Biodiversity Information Facility, Disease Vectors Database, the National Museum of Natural History (Smithsonian Institution) and published literature on *Lu. shannoni* surveillance and capture. Historical climate data from 1991–2020, along with projection data for Shared Socioeconomic Pathways 2–4.5 and 3–7.0 were obtained. An additional terrestrial ecoregions layer was applied. The ecological niche model was created using maximum entropy (MaxEnt) algorithms to identify regions which currently are or may become ecologically suitable for *Lu. shannoni*.

**Results:**

Currently, regions in eastern, western and southern Mexico, along with the Midwest, southeastern and eastern regions of the USA are ecologically suitable for *Lu. shannoni*. In the future, ecological suitability for *Lu. shannoni* is expected to increase slightly in the northeastern regions of the USA and in Atlantic Canada, and to decrease in the southeastern reaches of Mexico. Degree-days below 0 °C (spring and autumn), precipitation as snow (summer and winter), terrestrial ecoregions, number of frost-free days (summer), Hargreaves climatic moisture deficit (summer), degree-days above 5 °C (autumn) and Hogg’s climatic moisture index (summer) were all identified as predictors of ecological suitability.

**Conclusions:**

The findings from this study identified climate and environmental variables driving the ecological suitability of regions for *Lu. shannoni* and can be used to inform public health professionals of high-risk regions for exposure at present and into the future.

**Graphical abstract:**

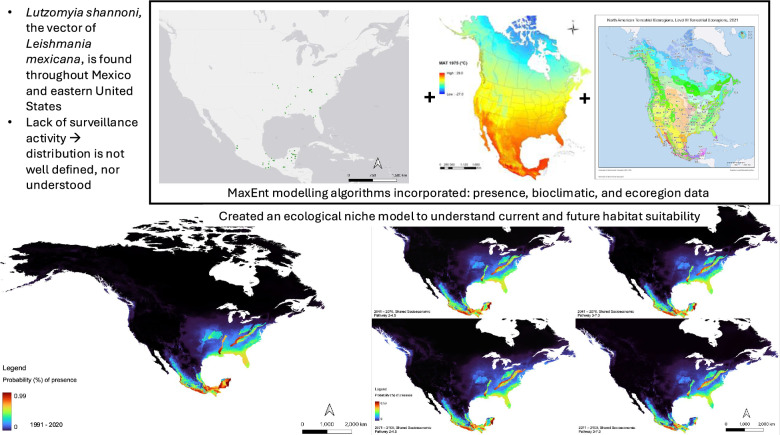

## Background

Phlebotomine sand flies, such as members of the *Lutzomyia* genus, are small (approx. 2 mm in size) pool feeders (i.e. blood-feeders that cut a hole in the skin and feed on the blood that pools from the wound) that are currently well-distributed in the Americas [1–10]. *Lutzomyia* spp. can be found in agricultural or rural areas of tropical and subtropical regions in countries such as the USA, Mexico, Guatemala, Belize, El Salvador, Nicaragua, Costa Rica, Ecuador, Colombia, Venezuela, Guyana, French Guiana, Suriname, Brazil, Argentina, Paraguay and Bolivia [1–4, 6, 8, 11]. Their life-cycle consists of four major stages: eggs, larvae, pupae and adults [1]. Female *Lutzomyia* spp. can live 2–6 weeks and undergo multiple gonotrophic cycles, and each cycle can produce anywhere from 30 to 70 eggs [1, 7, 8, 12, 13]. While adult sand flies consume plant sugars, female sand flies must also take multiple blood meals from mammals to produce eggs [1, 7, 8, 12, 13]. It is during these blood meals that *Lutzomyia* spp. females may ingest and then subsequently transmit—in a future meal—pathogens such as vesicular stomatitis virus (VSV) and *Leishmania* spp. to infective mammalian hosts [5, 7, 8, 14, 15].

VSV causes disease in horses, ruminants and other livestock species, including swine. Blister-like lesions form in the mouth, lips, ears, hooves and udders, causing discomfort and a significant reduction in productivity. Moreover, the lesions caused by VSV closely resemble those associated with foot and mouth disease virus and thus can result in pre-emptive quarantine and trade disruptions. VSV can also cause disease in humans [14, 15]. *Leishmania* spp. are the etiologic agent of leishmaniases in both humans and animals, namely dogs. Leishmaniasis is a global health issue that impacts approximately 2.5 million dogs and 12 million people, with over 700,000 new cases diagnosed annually [16, 17]. In humans, leishmaniasis can manifest as a visceral, cutaneous or mucocutaneous form, all of which are caused by different *Leishmania* spp. Visceral leishmaniasis (caused by *Leishmania infantum* and *Leishmania donovani*) is characterized by damage to the hosts’ internal organs, while cutaneous leishmaniasis (caused by *Leishmania mexicana, Leishmania braziliensis* and *Leishmania major*) causes skin lesions [16, 17]. Mucocutaneous leishmaniasis (caused by *L. braziliensis* and *Leishmania guyanensis*) causes damage to the mucous membranes of the host [14–16]. In dogs, infection with *L. infantum* or *L. braziliensis* is a multisystemic disease in nature, involving almost all the hosts’ body systems [16, 17, 19–24]. Common signs of infection include lymphadenopathy and dermatological abnormalities, with disease progression leading to renal failure and death [16, 17, 24]. Dogs also play a unique epidemiological role in *Leishmania* spp. transmission as they serve as reservoir hosts for *L. infantum* and *L. braziliensis*.

In North America, one of the northern-most sand flies, *Lutzomyia shannoni,* is a potential vector of VSV, along with *Leishmania* spp. *Lutzomyia shannoni* meets the requirements of a biological vector for the VSV, with vertical transmission having been demonstrated in laboratory studies and possibly in nature [14]. Further, laboratory studies have found *Lu. shannoni* to be capable of acquiring *L. mexicana* from infected animals and subsequently transmitting it during a subsequent blood meal [25, 26]. However, the relationship between the parasite and vector has not been confirmed in nature [25]. *Lutzomyia shannoni* has also been suspected to transmit *L. infantum,* as the former were reported in areas where *L. infantum*-infected dogs were reported, and *L. infantum* remains infective in *Lu. shannoni* following ingestion, although further confirmatory studies are lacking [27]. While current research suggests that *Lu. shannoni* demonstrate a lower infection rate compared to other sand flies, such as *Lu. longipalpis,* and infection has not been documented definitively in the USA, this sand fly remains a potential concern for domestic animal and human health as its range extends into more northern areas where other sand fly species are absent [27]. *Lutzomyia shannoni* has been found across 14 U.S. states, and as far north as the state of New Jersey [11, 15].

Over the past several decades, countries across the world have experienced climatic shifts due to anthropogenic carbon emissions and subsequent global warming [9, 10]. These climatic shifts have resulted in the expansion of ecological suitable areas for several vector species, such as ticks and mosquitoes [28–32]. It is unknown if sand flies such as *Lu. shannoni* may expand or shift their range under climate change and thus provide a mechanism for local transmission. This is particularly relevant as there is considerable international domestic animal movement, which could introduce VSV or *Leishmania* spp.

Ecological niche models (ENM) are statistical models that use environmental data to predict the distribution of a species (such as *Lu. shannoni*), based on ecological suitability [33–36]. These models can be utilized for a variety of reasons, such as when a species’ distribution is not well-defined and surveillance data are lacking. Maximum entropy (MaxEnt) algorithms are a type of ENM that utilize presence-only data [37]. As *Lu. shannoni* is an under-studied *Lutzomyia* sp., these models can be used to understand both their current potential distribution based on ecological factors, as well as regions of future ecological suitability, under different climate change scenarios.

Therefore, the objectives of this study were to (i) predict the current and future ecological suitability in North America for *Lu. shannoni,* and (ii) to identify variables impacting *Lu. shannoni* ecological suitability. As *Lu. shannoni* distribution is dependent on locally occurring ecological factors, such as precipitation, temperature and habitat availability (i.e. the presence of deciduous trees), such factors are expected to have considerable impact on ecological suitability both currently and into the future [15, 38, 39].

## Methods

### Study area

The terrestrial regions of North and Central America (as far south as Mexico) were utilized for ecological niche model generation. This extent captured the current distribution of *Lu. shannoni,* and the northward regions of interest for range expansion.

### Data acquisition and preparation

Presence-only data, including coordinates and year of collection, for *Lu. shannoni* were obtained from the Global Biodiversity Information Facility (GBIF) (https://www.gbif.org), the Disease Vectors Database (discontinued) and the National Museum of Natural History (Smithsonian Institution) (https://naturalhistory.si.edu). Additional presence points were obtained from previous surveillance data reported in the literature [2–4, 6, 11, 40–52]. The distance between presence points was investigated in QGIS version 3.22.1 (https://qgis.org/en/site; 2024). To reduce artificial clustering and spatial bias correction, all duplicate presence points and points less than 3.5 km apart were removed from further analyses [53].

Climate variables derived from daily weather data, such as temperature (minimum, maximum, average), precipitation values, frost-free days and degree days, were obtained from ClimateNA (1991–2020) (https://climatena.ca). Fifteen seasonal climate variables (60 variables total, one for each season) were downloaded at a resolution of 4 km^2^. An additional data layer, containing terrestrial ecoregions (level III), were obtained from the Commission for Environmental Cooperation (CEC; http://www.cec.org) (Table 1). Projection data were obtained from ClimateNA for emission scenarios based on socioeconomic predictions, known as Shared Socioeconomic Pathways (SSPs). The General Circulation Models (GCMs) for Shared Socioeconomic Pathways 2–4.5 and 3–7.0 (i.e. the ‘middle of the road’ and ‘upper middle’) were selected. SSP 2.4–5 assumes temperatures would rise by 2.7 °C by 2100, and SSP 3–7.0 assumes temperatures would rise by 3.6 °C by 2100 [54]. Data were projected into two 30-year periods: 2041–2070 and 2071–2100. Ecoregions remain stable over time; therefore, the same ecoregion layer was retained in both the base environmental layer and projection layer.

**Table 1:** Summary of environmental and bioclimatic variables investigated in *Lutzomyia shannoni* ecological niche model construction

Variable	Description	Included in final model?
CEC terrestrial ecoregions level III	Ecological regions are areas of similarity in ecosystems and environmental resources Level III ecoregions are smaller ecological areas within larger ecoregions	Yes
CMDsm	Hargreaves climatic moisture deficit (mm) [summer]	Yes
CMIsm	Hogg’s climate moisture index (mm) [summer]	Yes
CMIsp	Hogg’s climatic moisture index (mm) [spring]	No
DD0at	Degree-days below 0 °C [autumn]	Yes
DD0sm	Degree-days below 0 °C [summer]	No
DD0sp	Degree-days below 0 °C [spring]	Yes
NFFDsm	Number of frost-free days [summer]	Yes
NFFDsp	Number of frost-free days [spring]	No
NFFDwt	Number of frost-free days [winter]	No
NFFDat	Number of frost-free days [autumn]	No
PASat	Precipitation as snow [autumn]	No
PASsm	Precipitation as snow [summer]	Yes
PASsp	Precipitation as snow [spring]	No
PASwt	Precipitation as snow [winter]	Yes
PPTsm	Precipitation (mm) [summer]	No
PPTsp	Precipitation (mm) [spring]	No
PPTwt	Precipitation (mm) [winter]	No
Taveat	Mean temperature (°C) [autumn]	No
Tavesp	Mean temperature (°C) [spring]	No
Tavesm	Mean temperature (°C) [summer]	No
Tavewt	Mean temperature (°C) [winter]	No
Tmaxwt	Mean maximum temperature (°C) [winter]	No
Tmaxsp	Mean maximum temperature (°C) [spring]	No
Tmaxat	Mean maximum temperature (°C) [autumn]	No
DD5wt	Degree-days above 5 °C [winter]	No
DD5at	Degree-days above 5 °C [autumn]	Yes
DD5sp	Degree-days above 5 °C [spring]	No
DD18at	Degree-days above 18 °C [autumn]	No
DD18sp	Degree-days above 18 °C [spring]	No
Tminat	Mean minimum temperature (°C) [autumn]	No
Tminsp	Mean maximum temperature (°C) [spring]	No

*CEC* Commission for Environmental Cooperation

Data were imported into QGIS Version 3.22.1 to be rasterized. Data were geoprocessed to the same resolution and coordinate reference system (CRS84). Climate data at each presence point were extracted from QGIS and imported into RStudio (version 4.2.1; R Foundation for Statistical Computing, Vienna, Austria). A correlation matrix was created, and when highly correlated (> 0.80), retained variables were chosen based on ecological importance for *Lu. shannoni,* based on previous findings [10]. In the instance where > 1 highly correlated variable had ecological relevance, all were retained for initial model iteration.

### Ecological niche model—current projection

The initial ecological niche model was based on historic data. Presence data and rasterized ecological data were imported into MaxEnt species distribution modeling software (version 3.4.4) [53]. A *k-*fold cross-validation run-type was used to examine the data, where *k* = 4 [35, 37, 55–57]. Linear, quadratic and product feature classes were selected [35, 37, 55–57]. To prevent overfitting of the model, the regularization multiplier was increased [35, 37, 55–57] (Table 2). In previous research, the regularization multiplier has been changed from the default of 1.0, to values ranging from 1.5 to 3.0 [35, 37, 55–57]. A regularization multiplier of 1.0, 1.5 and 2.0 were applied, with the iteration returning the highest area under the receiver operating curve (ROC) (AUC) and lowest average omission rate being selected for projection layer application. The final model, with a regularization multiplier of 1.5, was chosen. An iterative approach was taken when building the ecological niche model, wherein the permutation importance of each variable was assessed. If a variable’s permutation importance was 0%, it would be removed from the model, and variables included in the following iteration had an importance of > 0%. When highly correlated variables were included in the final model, the correlated variable with the higher permutation importance was retained, while the other was removed. The final model was comprised of variables with a permutation importance of > 0%, and no highly correlated variables. Independent response curves for each environmental variable, along with a jackknife test of regularized training gain were generated.

**Table 2:** Initial maximum entropy model parameter settings applied in ecological niche model construction to investigate ecological suitability for *Lutzomyia shannoni* in North America

Parameters	Parameter setting
Feature class(es)	Linear, quadratic, product
Output	Clog log
Regularization multiplier	1.5
Replicated run type	*k*-fold cross-validation
*k*	4

### Ecological niche model—future projections

Variables included in the final current ecological niche model were considered to have an impact on *Lu. shannoni,* and therefore carried forward when generating future projection maps. No retained variables were highly correlated.

### Model evaluation

Model fit was evaluated using the mean AUC and the test omission rate of the minimum training presence. The model returning the highest AUC (i.e. closest to 100%) and lowest omission rate (i.e. closest to 0) was the best fitting model and therefore considered to be the final ecological niche model.

## Results

### Vector species records

**Fig. 1:**
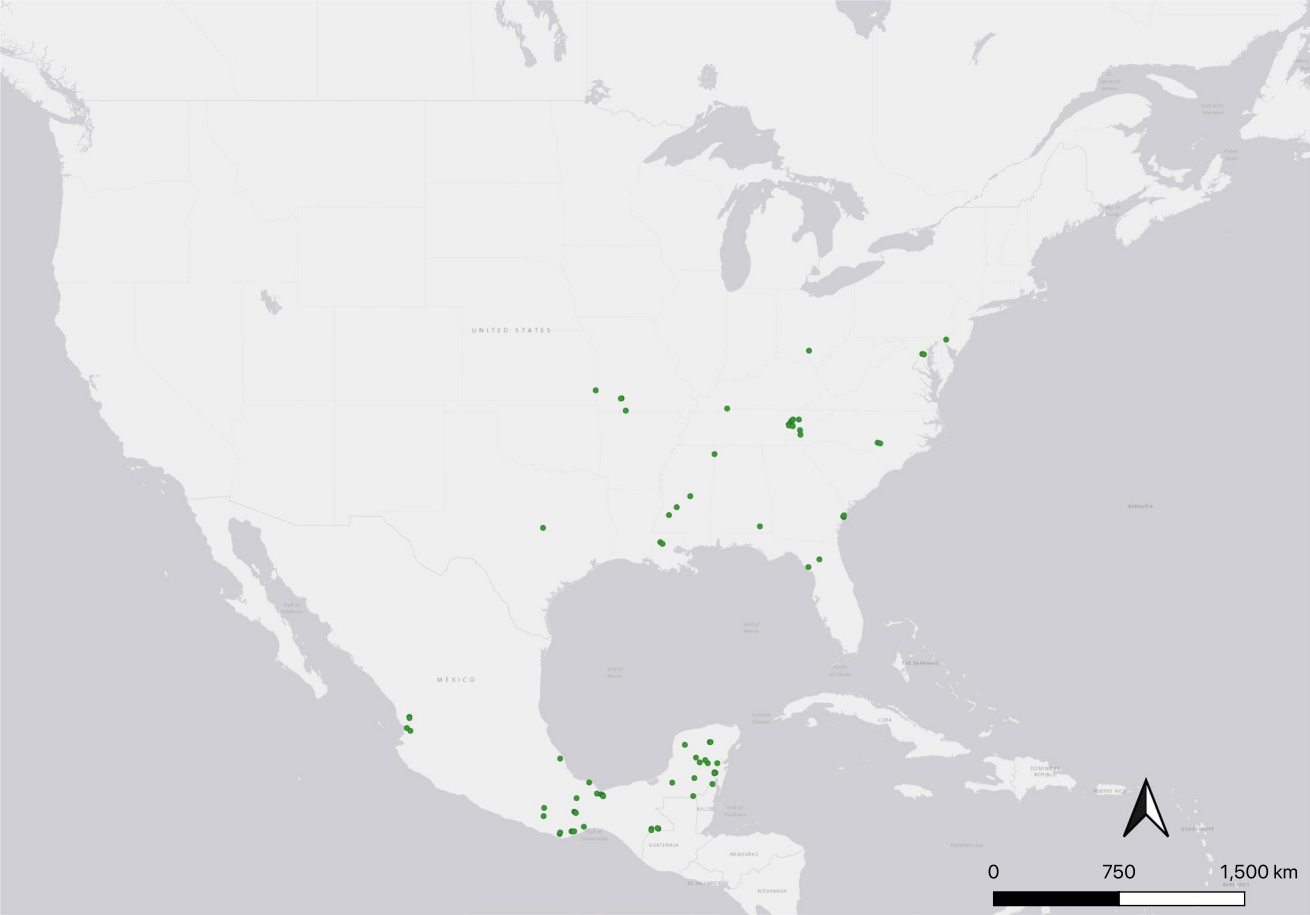
The geographic location of presence points for *Lutzomyia shannoni* (*n* = 80). Presence points were identified on the basis of human or machine observation, from 1991 to 2020. Map was constructed in QGIS (version 3.22.1)

Following the removal of duplications and rarefaction of coordinates, 80 presence points from 1991 to 2020 were eligible for inclusion. Species records were collected as far south as the states of Oaxaca and Veracruz, Mexico. The most northern records were collected from the U.S. states of Ohio, Maryland and New Jersey (Fig. 1).

### Ecological niche model

**Fig. 2:**
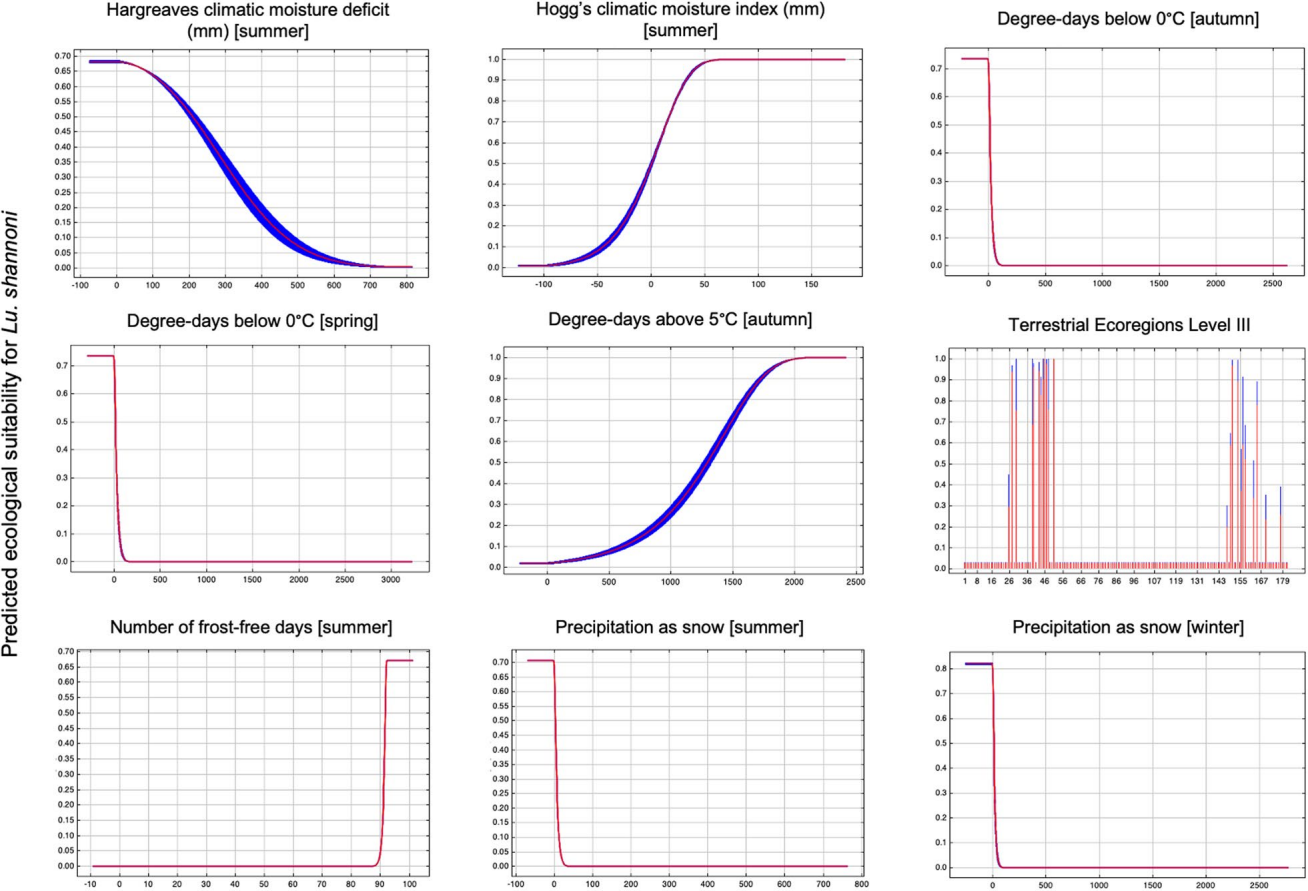
Independent response curves depicting the dependence of predicted ecological suitability for *Lutzomyia shannoni* on each modeled environmental variable. The red bands indicate the mean response, and the blue bands indicate the standard deviation. Graphs were constructed using the maximum entropy (MaxEnt) (version 3.4.4) algorithms [56]

Terrestrial ecoregions and 31 bioclimatic variables were included in initial model building [10] (Table 1). After removing all variables with a permutation importance of 0, no remaining variables were highly correlated. The final model predicting the ecological suitability of *Lu. shannoni* in North America included degree-days below 0 °C (autumn) (permutation importance of 73.6%), precipitation as snow (summer) (8.0%), precipitation as snow (winter) (5.3%), degree-days below 0 °C (spring) (4.7%), CEC terrestrial ecoregions level III (2.4%), number of frost-free days (summer) (2.3%), Hargreaves climatic moisture deficit (summer) (1.8%), degree-days above 5 °C (autumn) (1.2%) and Hogg’s climatic moisture index (summer) (0.6%) (Fig. 2; Tables 2, 3).

**Table 3:** Summary of maximum entropy outputs for the final ecological niche model

Environmental variable	Permutation importance (%)	Area under the receiver operating characteristic curve	Mean test omission rate (minimum training presence)
CEC terrestrial ecoregions level III	2.4		
Hargreaves climatic moisture deficit (summer)	1.8		
Hogg’s climatic moisture index (summer)	0.6		
Degree-days below 0 °C (spring)	4.7		
Degree-days below 0 °C (autumn)	73.6	97.4% ± 0.003	0.027
Degree-days above 5 °C (autumn)	1.2		
Number of frost-free days (summer)	2.3		
Precipitation as snow (winter)	5.3		
Precipitation as snow (summer)	8.0		

*CEC* Commission for Environmental Cooperation

**Fig. 3:**
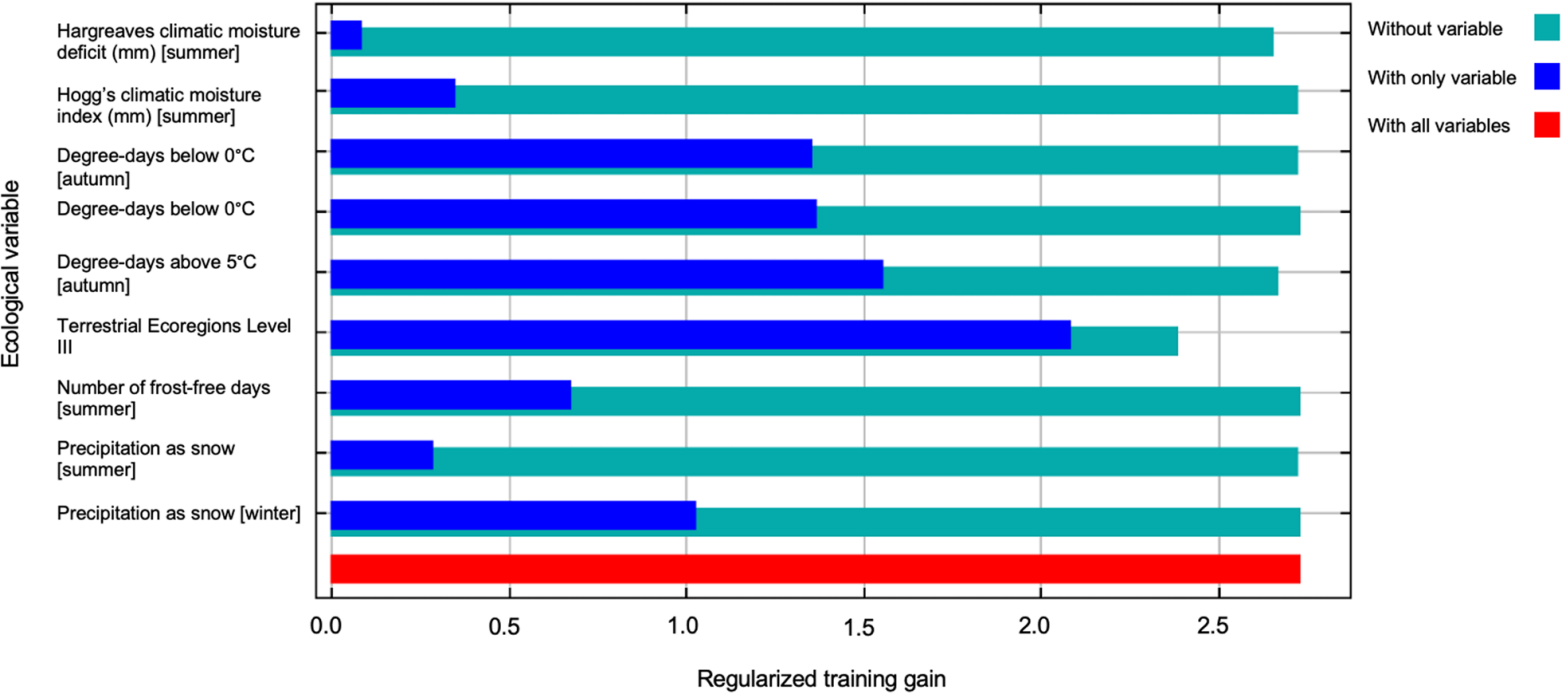
Jackknife test of variable importance for *Lutzomyia shannoni* ecological niche model. This test determines the regularized training gain of each variable in the final model, demonstrating which variables have the greatest impact on model gain when in isolation (indicated in blue), or when omitted from the model (indicated in teal). Graph was constructed using the maximum entropy (MaxEnt) (version 3.4.4) algorithms [56]

The model had an AUC of 97.4%, with a standard deviation (SD) of ± 0.003. According to Fielding and Bell [55], this AUC value was indicative of a good model fit. Using the mean minimum training presence of the test data, we determined the omission rate to be 0.027 (Table 3). Based on the independent response curves of the ecological variables, suitability for *Lu. shannoni* increased when the Hogg’s climatic moisture index in the summer, degree-days above 5 °C in the autumn and the number of frost-free days in the summer increased. Additionally, regions ecologically suitable for *Lu. shannoni* decreased when there were increases in the degree-days below 0 °C in the spring and autumn, Hargreaves climatic moisture deficit in the summer and precipitation as snow in the summer and winter. Some terrestrial ecoregions were associated with an increase in ecological suitability (i.e. predicted ecological suitability of > 0.70), including the Mississippi valley loess plain; ridge and valley; southern coastal plain; Sierras of Guerrero and Oaxaca with conifer, oak and mixed forests; Chiapas highlands with conifer, oak and mixed forest; south Pacific hills with and piedmonts with low tropical deciduous forest; Gulf of Mexico coastal plain with wetlands and high tropical rain forest; hills with medium and high evergreen tropical forest; plain with low and medium deciduous tropical forest and hills with high and medium semi-evergreen tropical forest; Los Tuxtlas Sierra with high evergreen tropical forest; and Jalisco/Nayarit hills and plains with medium semi-evergreen tropical forest . Results of the jackknife test of variable importance indicated that the variable with the highest independent gain was the CEC terrestrial ecoregions level III layer. Further, when this variable was omitted, the model gain decreased the most (Figs. 2,3).

#### Predicted current ecological suitability, 1991–2020

**Fig. 4:**
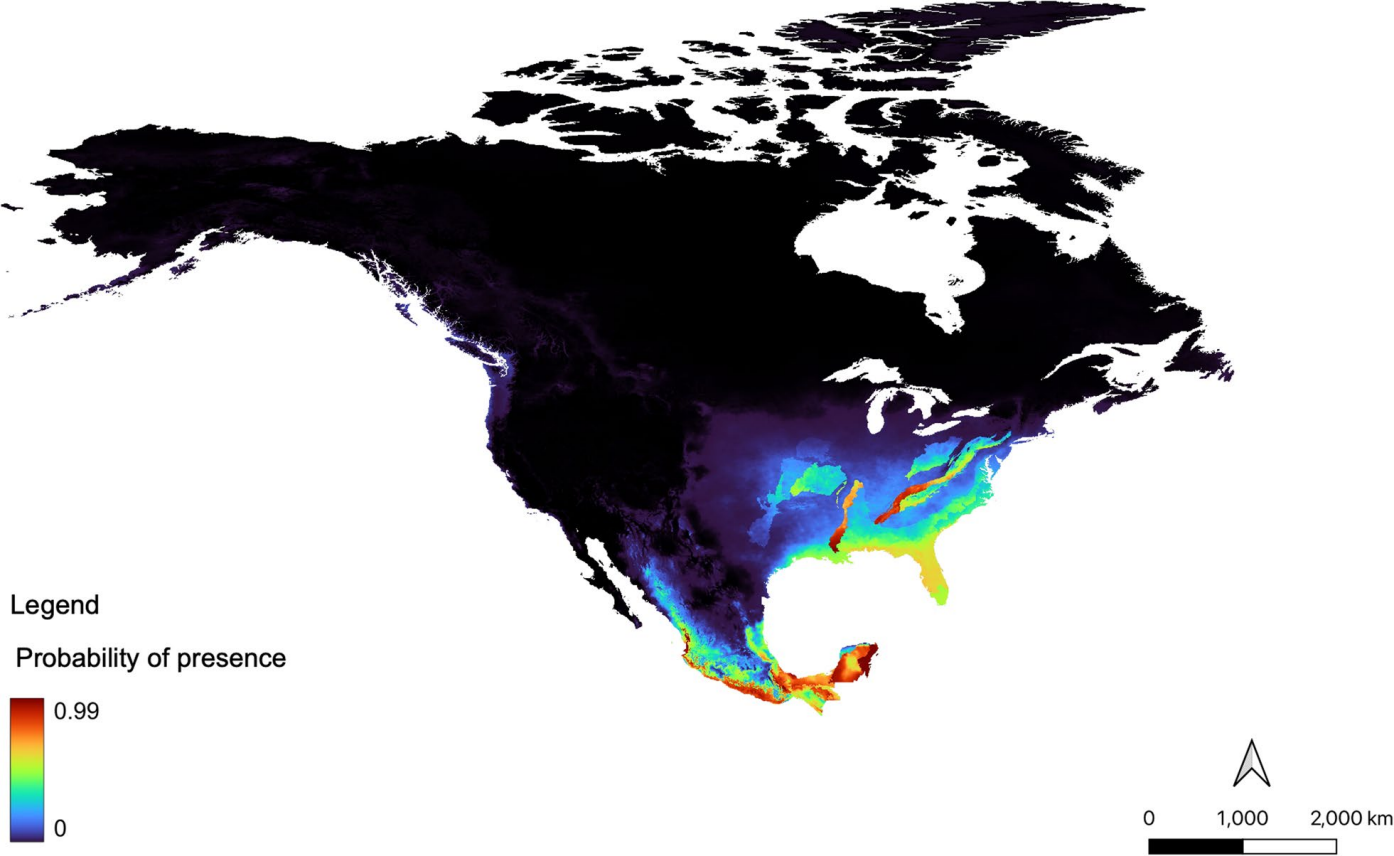
Current ecological suitability model for *Lutzomyia shannoni*, using climate and ecoregion data from 1991–2020. Warm colors indicate areas of high suitability, whereas cool colors are indicative of areas with low suitability. Map was constructed using the maximum entropy (MaxEnt) (version 3.4.4) algorithms [56]

Regions predicted to be currently ecologically suitable for *Lu. shannoni* in Mexico included the majority of southern Mexico, including both eastern and western extents. Much of central and northern Mexico is not currently ecologically suitable for *Lu. shannoni* . In the USA, suitable regions were across the Midwest, southeastern and eastern states. The west coast of the USA was not ecologically suitable for *Lu. shannoni*. In Canada, the coastal region of the province of British Columbia was found to have low ecological suitability. Terrestrial ecoregions suitable for *Lu. shannoni*, from 1991 to 2020, were variable in their characteristics, but could be generally defined by their high humidity and forest cover, specifically, deciduous and tropical forest types (Fig. 4).

#### Projected future ecological suitability, 2041–2070

##### Shared socioeconomic pathway 2–4.5

**Fig. 5:**
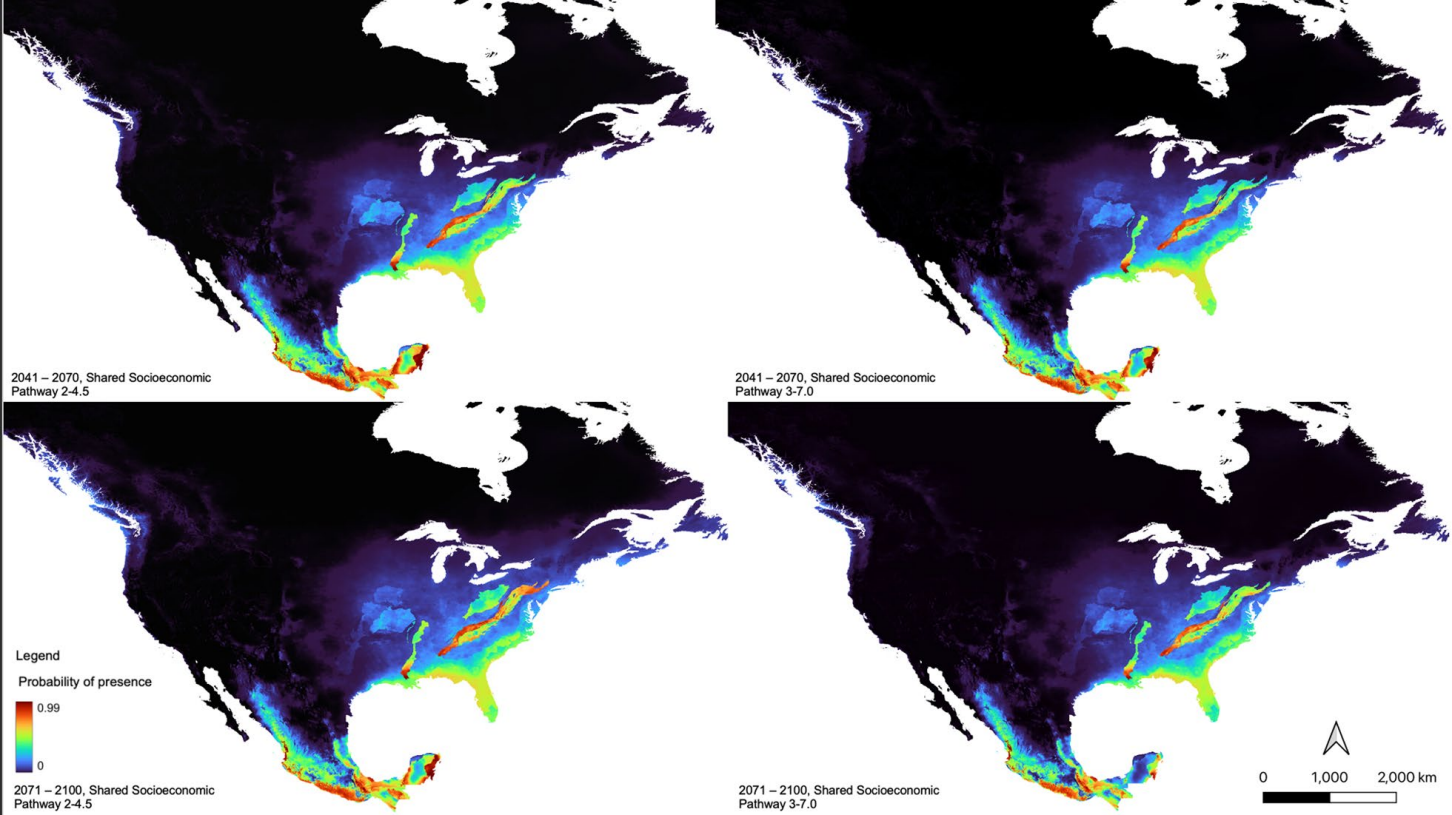
Future ecological suitability model for *Lutzomyia shannoni,* from 2041 to 2070, and from 2071 to 2100, under shared socioeconomic pathways (SSP) 2–4.5 and 3–7.0. Warm colors indicate areas of high suitability, and cool colors are indicative of areas with low suitability. Map was constructed using the maximum entropy (MaxEnt) (version 3.4.4) algorithms [56]

Based on this climate projection, there was a constriction of ecological suitability in southeastern Mexico and in the Midwest of the USA. Western Mexico and northeastern regions of the USA are forecasted to expand in their ecological suitability for *Lu. shannoni* (Fig. 5).

##### Shared Socioeconomic Pathway 3–7.0

With this climate projection, ecological suitability for *Lu. shannoni* decreased along the southeastern regions of Mexico, and slightly decreased in the Midwest of the USA. There was a slight increase in ecological suitability noted again in the western regions of Mexico, as well as along the southeastern and northeastern regions of the USA (Fig. 5).

#### Projected future ecological suitability, 2071–2100

##### Shared socioeconomic pathway 2–4.5

The largest general expansion of ecological suitability was observed with this climate projection. Under SSP 2–4.5, constriction in southeastern Mexico and the Midwest of the USA was noted again. Regions such as western Mexico, northeastern USA, Atlantic Canada and coastal British Columbia were all forecasted to increase in terms of their ecological suitability for *Lu. shannoni* (Fig. 5).

##### Shared socioeconomic pathway 3–7.0

This projection forecasted considerable constriction across southern and southeastern Mexico, and again in the Midwest of the USA. Ecological suitability for *Lu. shannoni* was forecasted to increase in northeastern regions of the USA (Fig. 5).

## Discussion

The ongoing impacts of climate change on ecological niche shifts of many dipteran vectors have been well-documented [30, 31]. Many of these dipterans are capable of transmitting pathogens infective to humans and animals, and *Lutzomyia* spp. are no exception. Continuous investigation into the ecological niche of *Lutzomyia* spp., including *Lu. shannoni*, is important to gain an understanding of which regions are currently suitable, and which could become suitable under changing climate conditions. In this study, we elucidated information on the current and future ecological suitability of North America for *Lu. shannoni*, a potential vector of VSV, *L. mexicana* and *L. infantum* [2, 7, 15, 20]. In the model utilized, ecological suitability was greatly impacted by degree-days below 0 °C (autumn), precipitation as snow (summer and winter), degree-days below 0 °C (autumn) and, to a lesser extent, terrestrial ecoregions, number of frost-free days (summer), Hargreaves climatic moisture deficit (summer), degree-days above 5 °C (autumn) and Hogg’s climatic moisture index (summer). Under the investigated climate scenarios, there were slight shifts in suitability across southern Mexico, and additional shifts across the eastern USA, coastal British Columbia and Atlantic Canada.

Degree-days below 0 °C (autumn) and terrestrial ecoregions had the highest permutation importance and greatest gain in the model, respectively. Therefore, it can be inferred that shifts in ecological suitability for *Lu. shannoni* are largely driven by these two variables. Degree-days are the number of days when the temperature is above or below a fixed reference value, typically with reference to vector development [58]. This parameter has been long identified as a major driver for vectors, namely due to its impact on vector activity and development [58]. In the context of this research, fewer degree-days below 0 °C in the autumn and spring were positively associated with ecological suitability. Further, ecological suitability increased with increasing number of degree-days above 5 °C in the autumn. Indeed, warmer transitional seasons (spring and autumn) would provide more developmentally favorable conditions for *Lu. shannoni* [5, 8–10, 59, 60]. Degree-days are a temperature-dependent variable, and the importance of temperature for *Lutzomyia* spp. development and activity has been well documented. For example, previous studies have reported that temperatures above 15 °C are ideal for *Lutzomyia* spp., but other studies have reported that minimum temperatures need to remain above 10 °C for at least 3 months for *Lutzomyia* spp. to become established [7, 10, 61].

 Terrestrial ecoregions are ecological regions with general similarities between their ecosystems, including the environmental resources they possess [62]. The CEC terrestrial ecoregions (level III) are smaller ecological areas nested into larger ecoregions [63, 64], allowing for the elucidation of more specific habitat information. Information on the ecoregion requirements for *Lu. shannoni* is fundamental to determining their ecological niche. Ecoregions provide distinct boundaries, allowing for extrapolation from presence points to whole ecoregions. Additionally, the common characteristics defining ecoregions provide additional ecological information regarding the niche of *Lu. shannoni.* In this study, numerous ecoregions were identified as ecologically suitable for *Lu. shannoni,* being defined by generally warm, temperate climates with a mix of deciduous, evergreen and tropical forests. Specifically, it is known that adult *Lu. shannoni* can be found in tree holes of deciduous trees [15, 38]. Therefore, ecoregions with this vegetation present likely serve as refugia sites. Despite the clear importance of ecoregions when elucidating the ecological niche of these sand flies, they have been rarely incorporated into ecological niche models of other *Lutzomyia* spp. [5, 9].

Due to the sensitivity of *Lu. shannoni* to temperature, it is known that frost and snow negatively impact their development [65, 66]. From the model, an increase in snow or frost was associated with a decrease in ecological suitability. Both snow and frost can only form at low temperatures, specifically those of less than 0 °C [65, 66]. As stated above, it is known *Lu. shannoni* require temperatures consistently higher than 0 °C for establishment to occur [7, 10, 61]. While the presence of snow and frost has negative impacts on *Lu. shannoni*, these factors are also an extension of temperature, specifically low ones, that are not conducive to their development, activity and potential establishment. Further, *Lu. shannoni* are small (2 mm) and considered to be weak fliers [1–10]. Therefore, snow presents a physical issue for *Lu. shannoni*, which are known to be sensitive to both wind and heavy precipitation.

Hargreaves climatic moisture deficit and, to a lesser extent, Hogg’s climatic moisture index (in the summer) were also found to be important when determining the ecological niche of *Lu. shannoni,* albeit to a lesser extent than degree-days and terrestrial ecoregions. Hargreaves climatic moisture deficit is calculated as potential evapotranspiration against actual evapotranspiration [67]. This measurement is strongly correlated with the distribution of vegetation in a landscape, and the moisture deficit accumulates over the season. The model generated in this study reported an inverse relationship between the moisture deficit and ecological suitability, with an increase in the moisture deficit being associated with a decrease in ecological suitability for *Lu. shannoni* [67]. Hogg’s climatic moisture index is an indicator of drought, with positive values being indicative of wet or moist conditions able to sustain a close-canopy forest [64]. In this ecological niche model, a higher moisture index was associated with an increase in ecological suitability. The importance of precipitation and moisture in the ecological niche of *Lutzomyia* spp. sand flies is well established [5, 8–10]. It is known that environmental moisture supports *Lutzomyia* spp. development, along with the creation of refugia [5, 8–10].

Based on the results from the presence records that were obtained, *Lu. shannoni* is currently found throughout southern and central Mexico and into central and eastern USA. The results from our ecological niche model align well with the known distribution of *Lu. shannoni* but they did identify some regions that are currently ecologically suitable, but from which no records have been recorded. These regions include coastal British Columbia and some parts of western Mexico and northeastern USA. Some regions of constriction and expansion were noted in the future projections. Generally, model projections predicted a decrease in ecologically suitable habitats across southeastern regions of Mexico and the Midwest regions of the USA, with many regions in Mexico and the USA forecasted to remain ecologically suitable. In Canada, regions such as coastal British Columbia and some reaches of the Maritimes were forecasted to become suitable, albeit only slightly.

It is important to note that even if areas are deemed currently ecologically suitable, *Lu. shannoni* require a dispersal mechanism to facilitate range shifts (e.g. human intervention, extreme weather events, among others). The data utilized in ecological niche models are not suitable to investigate specific dispersal mechanisms. For example, previous research has implicated wind-borne incursions of other vectors, such as biting midges (*Culicoides* spp.) and black flies (*Simulium* spp.), across 600–700 km [68, 69]. The relevance of wind for dispersal remains unknown for *Lu. shannoni*, since high winds can lead to a subsequent reduction in activity [68, 69]. Regardless, wind speed is associated with local weather conditions occurring within defined short periods of time and is not a specific component of larger scale and longer timeframe climate data utilized in ENMs. Further, physical barriers, such as the Rocky Mountain range that spans from western Canada to the southwestern USA, would need to be considered as they may interfere with dispersal. This is particularly relevant since coastal British Columbia is forecasted to be suitable area for *Lu. shannoni* in the future. However, given there are no known *Lu. shannoni* populations in any proximity to this region and the Rocky Mountains create a large physical barrier, dispersal via natural mechanisms is unlikely [7, 8].

There is a notable lack of continuous surveillance for *Lu. shannoni* across Mexico and the USA. Ecological suitability for the model was inferred using presence-only data, and given limited surveillance, it is probable that *Lu. shannoni* are present in other regions not incorporated into the model. While increasing the regularization multiplier of the model can reduce the risk of over-fitting the model, it is possible that additional suitable regions for *Lu. shannoni* exist (based on what is known of their ecology). Despite methodological adjustments for the sample size (i.e. setting a regularization multiplier of 1.5, *k-*fold cross-validation), sample size was still relatively small (*n* = 80), which introduces uncertainty and the possibility of model underfitting in some regions while overestimating regions of suitability in others [33, 35, 37, 55–57]. That being said, the high AUC and low mean omissions rate indicate the model is well fitted to the data [33, 35, 37, 55–57].

Regardless, it is important for public health professionals to be aware of current regions throughout Mexico and the USA within the ecological niche of *Lu. shannoni* described here. Due to the vector potential of this sand fly, and its implications for the health of humans, companion animals and livestock, it is recommended that surveillance efforts be concentrated to these highly suitable regions. In the USA and Canada, dogs are imported from *Leishmania-*endemic countries regularly [70–75]. In some instances, infected dogs are imported into Canada [72, 75]. While leishmaniasis cases in humans are rare in the USA and Canada, *Lu. shannoni’*s significance as a potential vector of *L. mexicana* and *L. infantum,* along with VSV in livestock, should encourage surveillance in currently suitable regions to monitor range shifts.

## Conclusions

The ecological niche models generated through this study provide important insights into regions of current and future suitability for *Lu. shannoni* across North America. Surveillance efforts should be directed at these regions to monitor populations and potential range shifts given the public and animal health relevance of this vector. That being said, ecological niche models cannot predict range expansion, and while regions may be identified as being suitable now or in the future for *Lu. shannoni,* future research is needed to explore factors related to dispersal, such as human and animal travel or extreme weather events.

## Data Availability

Species presence data were obtained from the Global Biodiversity Information Facility (GBIF), the Disease Vectors Database (discontinued) and the National Museum of Natural History (Smithsonian Institution). Additional presence points were obtained from previous surveillance data reported in the literature. Bioclimatic variables were obtained from ClimateNA, and an additional ecoregion layer was obtained from the Commission for Environmental Cooperation.
