# Publisher Correction: Assessing current and future areas of ecological suitability for *Lutzomyia shannoni* in North America

**DOI:** 10.1186/s13071-025-06848-2

**Published:** 2025-06-10

**Authors:** Sydney DeWinter, Grace K. Nichol, Christopher Fernandez-Prada, Amy L. Greer, J. Scott Weese, Katie M. Clow

**Affiliations:** 1 Department of Population Medicine, Ontario Veterinary College, University of Guelph, Guelph, ON Canada; 2 Department of Pathology and Microbiology, Faculty of Veterinary Medicine, University of Montreal, Saint-Hyacinthe, QC Canada; 3 Department of Biology, Trent University, Peterborough, ON Canada; 4 Department of Pathobiology, Ontario Veterinary College, University of Guelph, Guelph, ON Canada

**Publisher Correction: Parasites & Vectors (2025) 18:154** 10.1186/s13071-025-06781-4

Following publication of the original article [1], it came to the journal's attention that was an error in the graphical abstract. Namely, not all of the text in the image was readable. The image has since been update with a corrected version in which all the text is readable. Thank you for reading this erratum.
